# Development and validation of a new predictive model for macrosomia at late-term pregnancy: A prospective study

**DOI:** 10.3389/fendo.2022.1019234

**Published:** 2022-11-17

**Authors:** Yuhan Wang, Hongzhou Liu, Jincheng Wang, Xiaodong Hu, Anning Wang, Zhimei Nie, Huaijin Xu, Jiefei Li, Hong Xin, Jiamei Zhang, Han Zhang, Yueheng Wang, Zhaohui Lyu

**Affiliations:** ^1^ Department of Endocrinology, The First Medical Center, Chinese People’s Liberation Army (PLA) General Hospital, Beijing, China; ^2^ Department of Endocrinology, First Hospital of Handan City, Handan, Hebei, China; ^3^ Department of Epidemiology, The George Washington University, Washington, DC, United States; ^4^ Department of Obstetrics, The Second Hospital of Hebei Medical University, Shijiazhuang, Hebei, China; ^5^ Department of Ultrasound Diagnosis, The Second Hospital of Hebei Medical University, Shijiazhuang, Hebei, China

**Keywords:** macrosomia, fetal growth, obesity, gestational diabetes mellitus, predictive model

## Abstract

**Objective:**

Fetal macrosomia is defined as a birth weight more than 4,000 g and is associated with maternal and fetal complications. This early metabolic disease may influence the entire life of the infant. Currently, macrosomia is predicted by using the estimated fetal weight (EFW). However, the EFW is inaccurate when the gestational week is gradually increasing. To assess precisely the risk of macrosomia, we developed a new predictive model to estimate the risk of macrosomia.

**Methods:**

We continuously collected data on 655 subjects who attended regular antenatal visits and delivered at the Second Hospital of Hebei Medical University (Shijiazhuang, China) from November 2020 to September 2021. A total of 17 maternal features and 2 fetal ultrasonographic features were included at late-term pregnancy. The 655 subjects were divided into a model training set and an internal validation set. Then, 450 pregnant women were recruited from Handan Central Hospital (Handan, China) from November 2021 to March 2022 as the external validation set. The least absolute shrinkage and selection operator method was used to select the most appropriate predictive features and optimize them *via* 10-fold cross-validation. The multivariate logistical regressions were used to build the predictive model. Receiver operating characteristic (ROC) curves, C-indices, and calibration plots were obtained to assess model discrimination and accuracy. The model’s clinical utility was evaluated *via* decision curve analysis (DCA).

**Results:**

Four predictors were finally included to develop this new model: prepregnancy obesity (prepregnancy body mass index ≥ 30 kg/m^2^), hypertriglyceridemia, gestational diabetes mellitus, and fetal abdominal circumference. This model afforded moderate predictive power [area under the ROC curve 0.788 (95% confidence interval [CI] 0.736, 0.840) for the training set, 0.819 (95% CI 0.744,0.894) for the internal validation set, and 0.773 (95% CI 0.713,0.833) for the external validation set]. On DCA, the model evidenced a good fit with, and positive net benefits for, both the internal and external validation sets.

**Conclusions:**

We developed a predictive model for macrosomia and performed external validation in other regions to further prove the discrimination and accuracy of this predictive model. This novel model will aid clinicians in easily identifying those at high risk of macrosomia and assist obstetricians to plan accordingly.

## Background

Macrosomia is defined as a birth weight more than 4,000 g and is one of the most common adverse neonatal outcomes worldwide. Macrosomia is strongly associated with severe adverse perinatal outcomes, including shoulder dystocia, maternal birth canal trauma, and fetal brachial plexus injury or fracture (1, 2). If the risk could be estimated more accurately, this would help reduce such outcomes (3). Several methods that were earlier developed to predict fetal birth weight remain in use in clinical practice. For example, the Hadlock formula for the estimation of fetal weight (EFW) uses fetal morphological ultrasonic or other parameters (4, 5). However, the American College of Obstetricians and Gynecologists recently reported that the accuracy of both the Hadlock formula and the formulae using clinical parameters to predict macrosomia were limited; this is because the EFW accuracy falls constantly as the gestational weeks increase, especially at late-term pregnancy (6). The use of EFW methods to predict macrosomia is associated with a high risk of incorrect delivery decisions (7, 8). A more accurate method is required. Some scholars have built predictive models to predict the newborn weight in recent years. However, these have certain limits. For example, some models are difficult to use in the clinic because they require seldom-measured fetal parameters, or some are applicable only to specific races (9–11). Moreover, the accuracy of these models has not been completely assessed and external validation evidence is lacking. In this study, we developed a novel predictive model and performed validations to identify patients at risk of delivering macrosomia easily, allowing rational intervention and appropriate prenatal decision-making.

## Methods

### Populations

**Figure 1:**
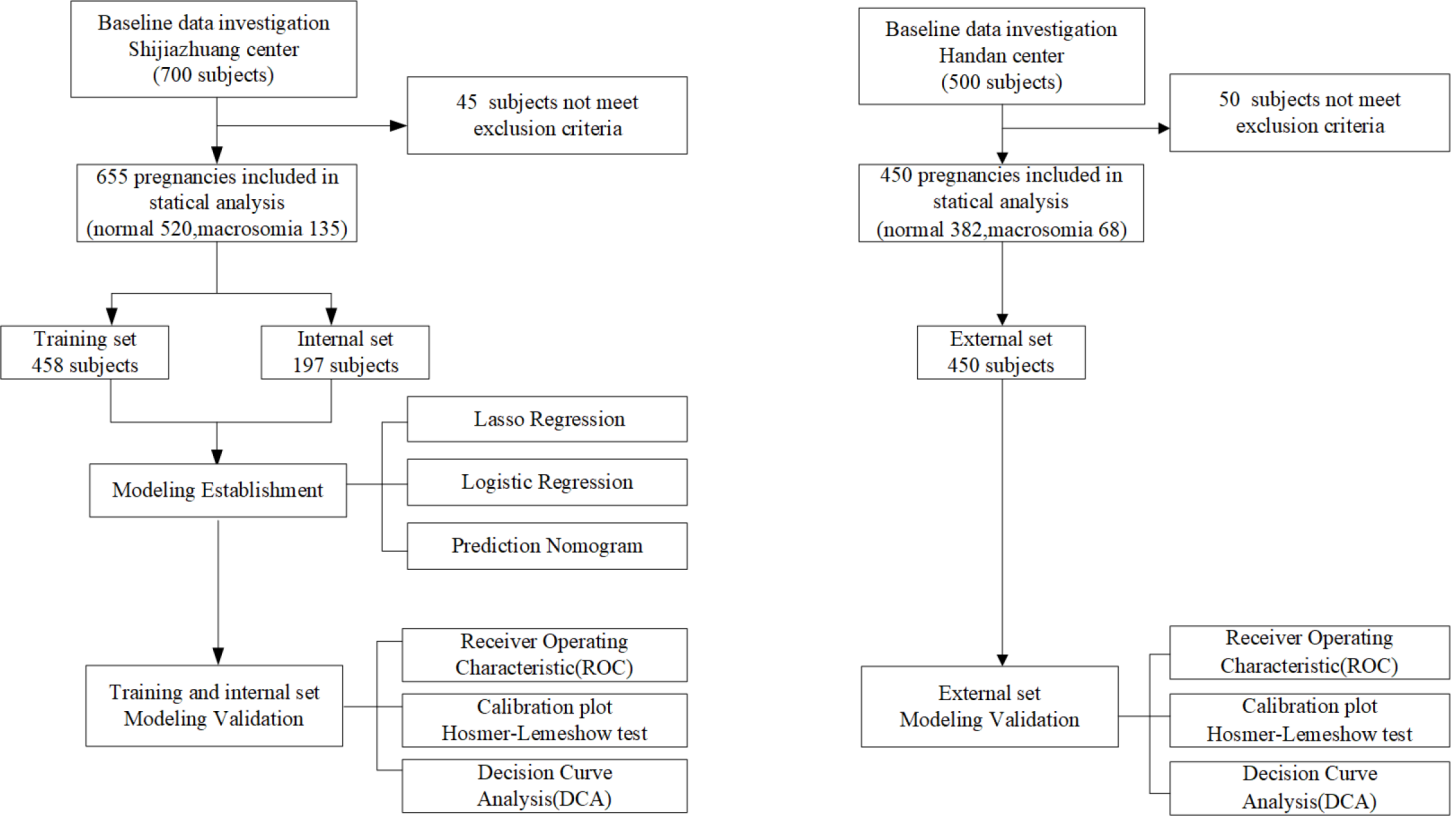
Study flow chart.

This is a prospective study. From November 2020 to September 2021, we prospectively recruited 700 pregnant women attending the Second Hospital of Hebei Medical University (Shijiazhuang, China) to conduct model development and internal validation. From November 2021 to March 2022, in another region, 500 pregnant women attending Handan Central Hospital (Handan, China) were prospectively recruited as the model’s external validation. All the data in two regions were continuously recorded in the primary healthcare systems. After excluding 95 patients (45 subjects from Shijiazhuang and 50 subjects from Handan) who did not meet the inclusion criteria, a total of 1,105 subjects were finally included in analysis. Based on the work of the two medical centers, we ultimately identified 19 relevant features, of which 17 were maternal features and 2 were fetal features. The flow chart of study design is shown in **Figure 1** (see **Figure 1**).

### Inclusion and exclusion criteria

The subject’s inclusion criteria were (1) maternal age ≥ 20 years; (2) a singleton pregnancy; (3) the completion of an oral glucose tolerance test (OGTT) at 24–28 weeks of gestation; and (4) a fetal ultrasound examination at 37–41 weeks of gestation. According to the World Health Organization (WHO), a body mass index (BMI) ≥ 30 kg/m^2^ reflects obesity. The diagnostic criteria for gestational diabetes mellitus (GDM) were those of the International Association of Diabetes and Pregnancy Study Groups (IADPSD): fasting plasma glucose (FPG) ≥ 5.1 mmol/L, oral glucose tolerance 1-h plasma glucose (OGTT 1hPG) ≥ 10.0 mmol/L, and oral glucose tolerance 2-h plasma glucose (OGTT 2hPG) ≥ 8.5 mmol/L on a 75-g OGTT test performed at 24–28 weeks of gestation; GDM was diagnosed when any of the three criteria were met (12). Hypercholesterolemia and hypertriglyceridemia were diagnosed using the criteria of the Guidelines for the American College of Cardiology/American Heart Association (13). The exclusion criteria were (1) multiple pregnancies; (2) gestational hypertension; (3) congenital heart disease; (4) a severe liver or kidney disease; (5) an autoimmune disease; (6) a psychiatric disorder; (7) the use of hormonal drugs during pregnancy; and (8) a fetal chromosomal abnormality or a congenital malformation. This study was approved by both Hebei Medical University and Handan Central Hospital.

### Predictive factors choose and measurements

Several candidate predictors were referred to previous studies. Other candidate predictors were obtained based on advice from experienced obstetricians, endocrinologists, and ultrasound physicians. Finally, 17 maternal and 2 fetal characteristics were included, which were proven to be potentially related with macrosomia: 1). maternal demographic characteristics: age, gestational weeks before delivery, maternal abdominal circumference, added weight during pregnancy, prepregnancy BMI, and uterine height at late-term pregnancy; 2). metabolic-related factors: the patient’s history of prepregnancy obesity, GDM, hypercholesterolemia, and hypertriglyceridemia during pregnancy; 3). biochemical features: the OGTT test results including the FPG, OGTT-1hPG, and OGTT-2hPG at gestational 24–28 weeks and the levels of low-density lipoprotein cholesterol (LDL-C), high-density lipoprotein cholesterol (HDL-C), serum creatinine, and serum uric acid at gestational 16 weeks; and 4). fetal growth parameters: the biparietal diameter and abdominal circumference at gestational 37–41 weeks. The blood biochemical tests concluded at gestational 16 weeks and OGTT tests concluded at 24–28 weeks of gestation. The prepregnancy BMI was calculated as the self-reported prepregnancy weight (kg)/height (m^2^) that was regularly registered in the patient’s primary healthcare systems. The added weight during pregnancy was calculated as the weight of an inpatient before delivery minus the self-reported prepregnancy weight (14). The uterine height was measured by an obstetrician *via* abdominal palpation at late-term pregnancy. A same measurement of fetal ultrasonographic parameters was performed according to the International Society of Ultrasound in Obstetrics and Gynecology (ISUOG) Practice Guidelines in two medical centers at a subject’s gestational 37–41 weeks (4): the subject lays supine or in the lateral position during examinations. A senior physician examined the fetus *via* three-dimensional abdominal ultrasonography and recorded the fetal ultrasonographic parameters. The ultrasound examinations were performed by an experienced ultrasound physician who was blinded to the study groups at the Second Hospital of Hebei Medical University and Handan Central Hospital.

### Outcome assessment

The weight of newborns was measured by nurses during the admission for delivery. All the newborns were weighed immediately after delivery by using the baby scale. Macrosomia was defined as a newborn weighing more than 4,000 g. The outcome measurement was completed by experienced obstetricians in two medical centers.

### Statistical analysis

A total of 655 patients from Shijiazhuang were randomly divided into a training set with 458 participants and an internal validation set with 197 participants with a 3:1 ratio. A total of 450 patients in Handan were analyzed for an external validation set. The t-test was used for analyzing numerical variables, and the Mantel–Haenszel chi-square test was utilized for analyzing categorical variables between groups. The method to achieve model selection is the last absolute shrinkage and selection operator (LASSO) regression method. The optimal penalty (lambda, λ) was estimated by using 10-fold cross-validation. According to the lambda-choosing path, the optimal penalty lambda could be present by the lambda with a minimum mean squared error (lambda.min) or the lambda.min with one standard error (lambda.1se) (15, 16). The univariable logistic regression was first used to evaluate the relationship between all the predictive features and the outcome. Then, to screen the potential optimal features, two multivariate logistic regression models with penalty was lambda.min (model 1) and lambda.1se (model 2) were built and compared to choose the most appropriate predictive features. The features were considered as odds ratio (OR) having 95% confidence interval (CI) and as a *P*-value. The statistical significance levels were all two sided. All of the selected features had statistical significance and were applied to develop the nomogram prediction models. The discriminatory ability of the model was evaluated by using receiver operating characteristic (ROC) curve analysis and C-indices. The accuracy of the model was evaluated by drawing the calibration curves, accompanied by using the Hosmer–Lemeshow test. The calibration curves were measured by the bootstrap method for 500 repetitions. Decision curve analysis (DCA) was used to determine the clinical practicability of nomograms based on the net benefit under different threshold probabilities. For sample size simulation, we used the formula to calculate the sample size required for developing the prediction model of a binary outcome recommended by Riley et al. (17). Missing values in the data sets were handled by using the multiple interpolation method. Statistical analyses were performed using R software (version 3.6.1; R Foundation for Statistical Computing, Vienna, Austria).

## Results

### Population characteristics

A total of 458 subjects were used to develop the model, 197 subjects were analyzed for internal validation, and 458 subjects were finally analyzed for external validation. The prevalence of macrosomia in the model training set (development set), internal validation set, and external validation set was 23%, 15%, and 15%, respectively. There was no significant difference between the training set and the internal validation set for all the 19 features. All the alternative features characteristics of three sets are listed in **Table 1** (see **Table 1**).

**Table 1:** Characteristics of the population in training set and validation sets.

Features	Training set(Shijiazhuang, n=458)	Internal Validation set(Shijiazhuang, n=197)	External Validation set(Handan, n=450)	*P*-value
Age(years)	31.21 ± 4.60	30.98 ± 4.58	28.29 ± 4.36	0.18
Gestational weeks at delivery (weeks)	38.29 ± 1.13	38.18 ± 1.05	38.46 ± 1.28	0.15
Prepregnancy BMI (kg/m^2^)	24.65 ± 4.57	24.05 ± 4.60	24.35 ± 4.70	0.13
Added weight during pregnancy (kg)	13.44 ± 2.37	13.21 ± 2.32	13.52 ± 2.39	0.24
Uterine height (cm)	33.29 ± 2.26	33.29 ± 2.18	32.08 ± 2.73	0.98
Maternal abdominal circumference (cm)	96.15 ± 7.31	95.33 ± 6.90	94.68 ± 7.01	0.07
GDM (%)				
Yes	210 (46)	76 (39)	116 (35)	0.09
No	248 (54)	121 (61)	334 (64)	
Prepregnancy obesity (%)				
Yes	131 (29)	51 (26)	116 (26)	0.48
No	327 (71)	146 (74)	334 (74)	
Hypertriglyceridemia (%)				
Yes	132 (29)	49 (25)	228 (38)	0.30
No	326 (71)	148 (75)	222 (62)	
Hypercholesteremia (%)				
Yes	141 (31)	67 (34)	103 (23)	0.42
No	317 (69)	130 (66)	347 (77)	
HDL-C (mmol/L)	1.52 ± 0.41	1.58 ± 0.40	1.83 ± 0.54	0.42
LDL-C (mmol/L)	3.11 ± 0.64	3.17 ± 0.63	3.05 ± 0.55	0.27
Serum creatinine (mmol/L)	62.26 ± 15.15	64.76 ± 15.35	67.81 ± 14.26	0.12
Serum uric acid (mmol/L)	294.06 ± 63.10	297.29 ± 64.87	292.44 ± 64.27	0.73
OGTT (mmol/L)				
FPG	4.88 ± 0.68	4.78 ± 0.62	4.93 ± 0.85	0.10
OGTT 1hPG	9.04 ± 1.38	9.02 ± 1.41	9.19 ± 1.71	0.93
OGTT 2hPG	6.89 ± 1.06	6.77 ± 1.11	6.95 ± 1.08	0.22
Fetal biparietal diameter (cm)	9.37 ± 0.36	9.42 ± 0.35	9.10 ± 0.61	0.09
Fetal abdominal circumference (cm)	35.03 ± 2.12	34.70 ± 2.20	35.09 ± 2.18	0.07

Values are expressed as means ± SD (standard variation) or frequency (%). GDM, gestational diabetes mellitus; HDL-C, high-density lipoprotein; LDL-C, low-density lipoprotein. OGTT, oral glucose tolerance test; FBG, fasting blood glucose; OGTT 1hPG, oral glucose tolerance 1-h plasma glucose; OGTT 2hPG, oral glucose tolerance test 2-h plasma glucose. P-values: Comparison between model development set and internal validation set by using the t-test or the Mantel–Haenszel chi-square test.

### Features selection and model development

**Figure 2:**
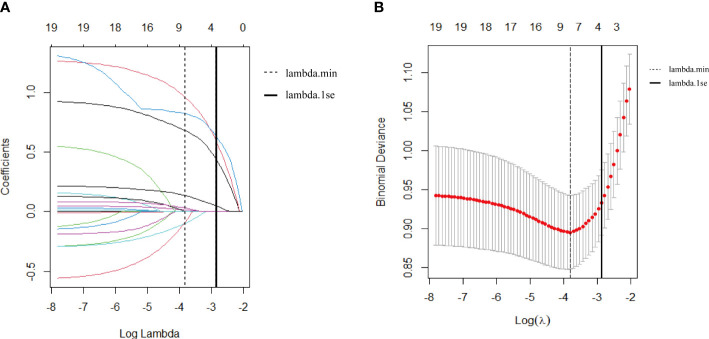
**(A)** Feature selection via the least absolute shrinkage and selection operator (LASSO) regression model. **(A)** The LASSO coefficient profiles of 19 features. The coefficient profile plot was conducted against the log (lambda, λ) sequence. The dotted vertical line was drawn at the lambda with a minimum mean squared error (lambda.min); nine features were selected by the LASSO regression. The solid vertical line was drawn at the lambda.min with one standard error (lambda.1se); four features were selected by the LASSO regression model. **(B)** Feature selection via 10-fold cross-validation. **(B)** The optimal parameter (lambda, λ) selection in the Lasso regression model used 10-fold cross-validation via the minimum criteria. The partial likelihood deviance (binomial deviance) curve was plotted versus logλ. The dotted vertical line was drawn at the lambda with a minimum mean squared error (lambda.min); nine features were selected. The solid vertical line was drawn at the lambda.min with one standard error (lambda.1se); four features were selected.

**Figure 3:**
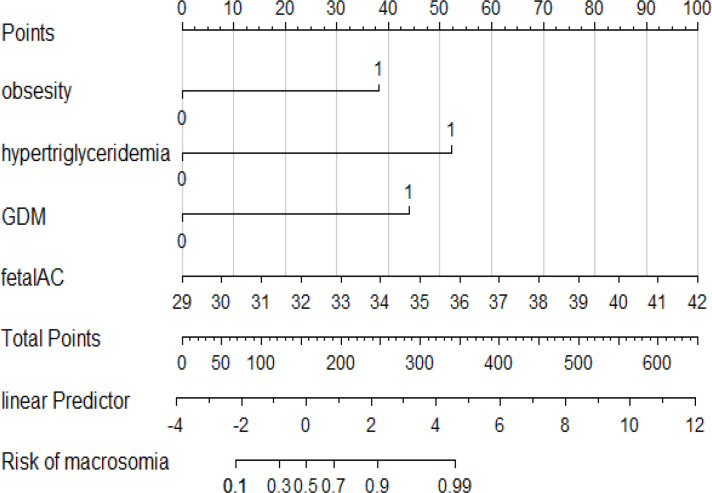
A nomogram prediction model of macrosomia. Four predictors were included: the prepregnancy obesity, hypertriglyceridemia, GDM, and fetal abdominal circumference. The score of each predictor were determined from each feature axis to the total points axis by following the vertical line. GDM, gestational diabetes mellitus; fetal AC, fetal abnormal circumference.

As shown in **Figure 2A**, we used LASSO regression to identify useful predictors from the 19 potential factors and then employed multivariate logistic regressions to build the model. In **Figure 2B**, nine features were saved under the optimal penalty that was lambda.min, and four features were finally saved under the penalty that was lambda.1se. **Table 2** shows the regression analysis of all the features. Model 1 (**Table 2**) shows that the multivariate logistic regression result with penalty was lambda. min. Model 2 (**Table 2**) shows that the multivariate logistic regression result with the penalty being lambda.1se. After comparing the results of two multivariate regression models, four features in Model 1 (**Table 2**) including the added weight, 2hPG, age, and gestational weeks were excluded as they were not significantly contributing to the outcome. Four features using lambda.1se were finally included to build the predictive model: the prepregnancy obesity (BMI ≥ 30 kg/m^2^), GDM, hypertriglyceridemia, and fetal abdominal circumference (**Table 2**). Then, we created a nomogram of macrosomia risk (See **Figure 3**). An example interpretation of this nomogram is as follows: a woman is not obese prepregnancy but develops GDM and hypertriglyceridemia during pregnancy, and the fetal abdominal circumference is 39 cm at 37–41 weeks of gestation. The latter three features attract the scores of 45, 52.5, and 77.5, respectively (total 175). The nomogram indicates that the risk of a macrosomia birth is almost 60%.

**Table 2:** Logistic regression analysis of the candidate predictors for macrosomia.

Candidate predictors	Univariate analysis			Multivariate analysis (Model 1)			Multivariate analysis (Model 2)		
	OR	95%CI	*P*-value	OR	95%CI	*P*-value	OR	95%CI	*P-*value
**Prepregnancy obesity**	4.09	2.75-6.08	<0.01	2.73	1.71-4.33	<0.01	2.54	1.51-4.27	<0.01
**GDM**	4.90	3.21-7.46	<0.01	2.40	1.30-4.42	0.01	2.95	1.49-6.03	<0.01
**Hypertriglyceridemia**	3.51	2.36-5.22	<0.01	3.04	1.89-4.89	<0.01	3.61	2.14-6.16	<0.01
**Fetal abdominal circumference**	1.46	1.31-1.62	<0.01	1.30	1.13-1.51	<0.01	1.21	1.02-1.43	0.03
**Added weight**	1.15	1.06-1.24	<0.01	1.04	0.94-1.14	0.50			
**OGTT 2hPG**	0.80	0.66-0.96	0.02	0.82	0.67-1.00	0.06			
**Age**	1.04	0.99-1.08	0.08	1.03	0.98-1.00	0.20			
**Gestational weeks**	1.20	1.02-1.40	0.02	1.12	0.93-1.36	0.24			
**Fetal biparietal diameter**	0.77	0.45-1.30	0.32	0.82	0.45-1.50	0.52			
**OGTT 1hPG**	1.49	1.49-1.72	<0.01						
**FBG**	2.29	1.71-3.08	<0.01						
**HDL-C**	1.06	0.78-1.45	0.71						
**LDL-C**	0.89	0.66-1.19	0.42						
**Serum uric acid**	1.00	0.99-1.00	0.32						
**Serum creatinine**	1.00	0.98-1.01	0.99						
**Uterine height**	1.03	0.95-1.12	0.47						
**Prepregnancy BMI**	0.97	0.93-1.01	0.11						
**Hypercholesteremia**	0.96	0.64-1.45	0.86						
**Maternal abdominal circumference**	1.03	1.00-1.06	0.04						

OR, odds radio; CI, confidence interval. GDM, gestational diabetes mellitus; FBG, fasting blood glucose; OGTT 1hPG, oral glucose tolerance test 1-h postprandial blood glucose. OGTT 2hPG, oral glucose tolerance test 2-h postprandial blood glucose. HDL-C, high-density lipoprotein cholesterol. LDL-C, low-density lipoprotein cholesterol. Model 1: Multivariate logistic regression with penalty was lambda. min based on LASSO and ten-fold validation test. Model 2: Multivariate logistic regression with penalty was lambda.1se based on LASSO and ten-fold validation test.

### Macrosomia risk factors

Four features were finally included to build the predictive model: prepregnancy obesity (95% CI 1.51,4.27 *p* < 0.001), GDM (95% CI 1.49,6.03 *P* = 0.002), hypertriglyceridemia (95% CI 2.14,6.16 *P* < 0.001), and fetal abdominal circumference (95% CI 1.02,1.43 *P* = 0.03) were independent risk factors for macrosomia (See **Table 2**, Model 2).

### Validation of the predictive model

**Figure 4:**
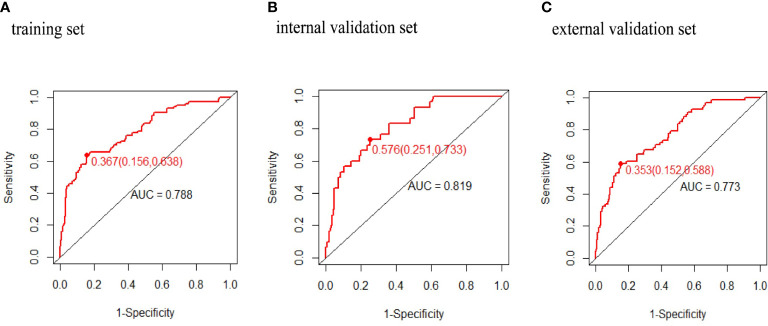
Receiver operating characteristic curves of macrosomia risk nomogram prediction. Receiver operating characteristic curve (ROC) of the **(A)** training set, **(B)** internal validation set, **(C)** external validation set. AUC, area under the receiver operating characteristic curve.

**Figure 5:**
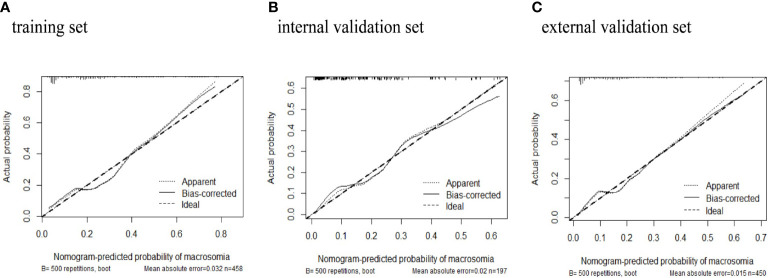
Calibration plots of macrosomia risk nomogram prediction. The x-axis represents the predicted risk of macrosomia. The y-axis represents the actual diagnosed case of macrosomia. The diagonal dotted line represents a perfect prediction by an ideal model. The solid line represents the performance of the **(A)** training set, **(B)** internal validation set, **(C)** external validation set. The closer fit of solid line to the diagonal dotted line represents a better prediction.

**Figure 6:**
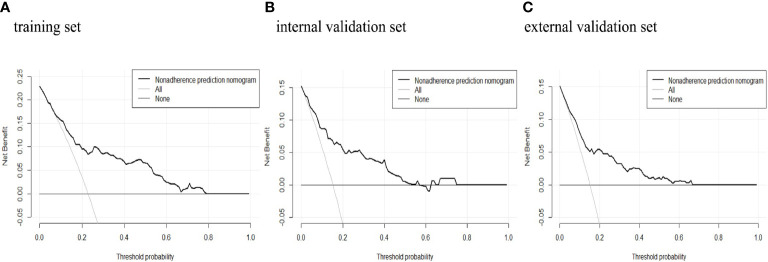
Decision curve analysis of macrosomia risk nomogram prediction. DCA of the **(A)** training set, **(B)** internal validation set, and **(C)** external validation set. The x-axis measures the threshold probability. The y-axis measures the net benefit. The thick, black solid line represents the macrosomia risk nomogram. The thin, black horizontal line (none line) represents the assumption that no patients are non-adherent to medication, which means that the net benefit is zero. The thin, gray bias (all line) represents the assumption that all patients are non-adherent to medication. DCA, decision curve analysis.

The predictive power was assessed by using the area under the ROC curves (AUC). The AUCs were 0.788 (training set), 0.819 (internal validation set), and 0.778 (external validation set) separately. The optimal cutoffs were 0.367 (training set), 0.576 (internal validation set), and 0.353 (external validation set) (see **Figure 4**). The C-indices were 0.788 (95% CI 0.736, 0.840), 0.819 (95% CI 0.744, 0.894), and 0.773 (95% CI 0.713, 0.833), respectively. The calibration plots of all three sets fit well with the ideal curves (see **Figure 5**). The Hosmer–Lemeshow test revealed that the predicted and actual probabilities were consistent (*P*_training set_ = 0.083, *P*_internal validation set_ = 0.762, *P*_external validation set_ = 0.074). We then used DCA to assess clinical utility (See **Figure 6**). The threshold probabilities of the model for the three sets were 3%–78%, 1%–57%, and 2%–66% respectively. As the incidence rate of macrosomia is reported to be 5.47%–31.3% in China (18, 19) and 8.07%–8.84% in other countries in literatures (20), DCA exhibited positive net benefits and potential clinical utility within these thresholds’ ranges (see **Figure 6**).

## Discussion

Macrosomia is strongly associated with multiple adverse perinatal outcomes in a previous study (21). Obstetricians and gynecologists have sought to improve screening; however, the predictive accuracy remains poor. In this study, we developed a predictive model applicable at late-term pregnancy to help guide the perinatal delivery strategy. Four simple predictors were finally selected as the most appropriate features to build this model: prepregnancy obesity (prepregnancy BMI ≥ 30 kg/m^2^), GDM, hypertriglyceridemia, and fetal abdominal circumference.

Metabolic features are strongly associated with fetal macrosomia. Prepregnancy obesity is one of the most common manifestations of metabolic dysfunction in different populations. For example, a prospective study on 912 Caucasians indicated that prepregnancy obesity increased the risk of macrosomia threefold (22). Another Asian study came to the same conclusion that pregnancies with prepregnancy obesity have a higher risk to give birth to macrosomia (23). In fact, obesity is accompanied by the manifestations of abnormal metabolism such as chronic inflammation, oxidative stress, and epigenetic changes; these may affect fetal growth *in utero* by compromising the placental function (24–26). Moreover, the constant high levels of circulating adipokines (leptin, adiponectin, and tumor necrosis factor-α) may impair insulin signaling, thus reducing maternal (and even fetal) insulin sensitivity, which may, in turn, affect fetal growth (27–31). Furthermore, the adipokine secretion levels in obese women differ from those in non-obese women, perhaps explaining the relationship between obesity and fetal macrosomia (32).

GDM was also associated with macrosomia. GDM is one of the most common metabolic diseases during pregnancy; the prevalence of GDM has gradually increased over recent decades (33). In a previous study, pregnant Asian women with GDM were at a higher risk of macrosomia than non-GDM women (34). The pathophysiological mechanism in play may be explained by the Pedersen hypothesis: GDM impairs maternal glycemic control; the serum glucose levels remain high, and then, more glucose crosses the placenta. Maternal or exogenously administered insulin does not cross the placenta. Thus, as glucose continuously crosses the placenta, compensatory hyperinsulinemia develops in the fetus (35). The risk imposed by GDM is thus twofold: not only does maternal metabolism become abnormal but also the fetus increases its adipose tissue and proprotein stores during growth, increasing the risk of macrosomia (36).

Abnormal lipid metabolism is another risk factor that may be associated with an offspring’s growth. Lipid levels do not change greatly during early pregnancy; however, from gestational week 12, intestinal fat absorption increases markedly, inducing physiological hyperlipidemia (37). In this study, we found that hypertriglyceridemia was a strong predictor of macrosomia, suggesting that abnormal lipid metabolism during pregnancy is closely linked to macrosomia. Hypertriglyceridemia during pregnancy raises the levels of plasma triglycerides and free fatty acids that enter the fetal circulation *via* the placenta, increasing fetal plasma protein synthesis and decreasing lipolysis; fetal lipids accumulate (38–40). Therefore, the control of maternal lipid levels (especially the triglyceride level) should be paid high attention to reduce the risk of macrosomia.

It is well known that antenatal ultrasonography valuably assesses the fetal intrauterine growth and detects fetal structural abnormalities that predict adverse pregnancy outcomes. The three-dimensional measurements of the biparietal diameter, the abdominal circumference, and the femoral length in late-term pregnancy can be used to derive the estimated fetal weight (EFW) (41). The question remains, which parameter is most closely related to macrosomia? Higgins et al. evaluated four common fetal ultrasonographic parameters commonly used to predict macrosomia in 416 pregnant women; their study suggested that the fetal abdominal circumference showed the highest predictive ability (42). In our model, we similarly found that the fetal abdominal circumference was the optimal predictor, especially during late-term pregnancy.

Several predictive models for macrosomia have been reported in previous studies (9, 11, 43, 44). For example, Mazouni et al. used a nomogram to predict macrosomia in 194 women (11). Their model included predictors as follows: the ultrasound-derived EFW at 37–42 weeks of gestation, parity, ethnicity, and the BMI. The AUCs of this model were 0.860 and 0.850 in the development set and internal validation set. The discrimination of their model was also better than that afforded by the Hadlock formula. However, this model was difficult to validate in Asians because one predictor, the race of subject, was limited to European, African, and Black in their study. Recently, Zou et al. developed a model to predict macrosomia for Asian GDM patients (9). This model includes the prepregnancy BMI, the gestational weight, the fasting plasma glucose and triglyceride levels, the fetal biparietal diameter, and the amniotic fluid index as predictors with the AUC of 0.813. However, the discrimination of Zou’s model is limited as the external validation is lacking. Their model was also confined to GDM subjects so that may not be fit to general pregnancies. Compared with the two previous models, the ROC curves of our model in the internal set and external set were 0.819 and 0.773, which suggested that the generalization ability of this novel model is certain. As the three maternal predictors in our model were both accessible at an earlier stage of pregnancy, the early prevention of metabolic-related factors may reduce the risk of macrosomia. During late-term pregnancy, this model could screen patients with a high risk of macrosomia and help clinicians to make correct delivery decisions for each patient.

### Study limitations

Although all the four predictors were easy to obtain in different populations, it should be noted that the model’s generalization ability needs more validation in different populations. We have referred to the international guidelines or recommendations to formulate the inclusion and exclusion criteria in this study. Thus, theoretically, the model is applicable to different races. Second, the timeframe of all the included features was formulated to be measured during pregnancy; however, the biochemical or ultrasound examinations may remain with several days’ (usually within 1 week) difference among the subjects due to patients’ personal reasons. This is common in the clinical practice but may still influence the accuracy of the nomogram. Despite its limitations, our study has the strength to prove the stable discrimination ability of this new model, such as the validation at different levels, well-organized sets, and the representative samples.

## Conclusion

We developed a nomogram that predicted macrosomia and confirmed both discrimination and accuracy *via* external validation. The key predictors were prepregnancy obesity, hypertriglyceridemia, gestational diabetes, and the fetal abdominal circumference. The model is easy to use and will assist obstetricians in terms of clinical decision-making.

## Data availability statement

The raw data supporting the conclusions of this article will be made available by the authors, without undue reservation.

## Ethics statement

The Institutional Ethics in Research Committee at the second hospital of the Hebei Medical University approved the study (2020-R-125). All participants provided written informed consent.

## Author contributions

YuHW wrote the manuscript. HL and JW contributed to data analyses. AW, HJX, and ZN collected the data used to develop this model. JL and XH collected the data used in validations. HZ and JZ performed the ultrasound examinations. YueHW and HX reviewed and revised the manuscript. ZL designed this study. All authors approved the final manuscript.

## Acknowledgments

The authors thank Xiaona Hu (The First Medical Center, Chinese PLA General Hospital) for the data collection work.

## Conflict of interest

The authors declare that the research was conducted in the absence of any commercial or financial relationships that could be construed as a potential conflict of interest.

## Publisher’s note

All claims expressed in this article are solely those of the authors and do not necessarily represent those of their affiliated organizations, or those of the publisher, the editors and the reviewers. Any product that may be evaluated in this article, or claim that may be made by its manufacturer, is not guaranteed or endorsed by the publisher.
